# Dopaminergic modulation of phase reversal in desert locusts

**DOI:** 10.3389/fnbeh.2014.00371

**Published:** 2014-11-07

**Authors:** Ahmad M. Alessi, Vincent O'Connor, Hitoshi Aonuma, Philip L. Newland

**Affiliations:** ^1^University College at Qunfudah, Umm al-Qura UniversityQunfudah, Makkah, Saudi Arabia; ^2^Centre for Biological Sciences, Faculty of Natural and Environmental Sciences, University of SouthamptonSouthampton, UK; ^3^Research Institute for Electronic Science, Hokkaido UniversitySapporo, Japan

**Keywords:** grasshopper, polyphenism, swarming, neural network, plasticity, dopamine

## Abstract

Phenotypic plasticity allows animals to modify their behavior, physiology, and morphology to adapt to environmental change. The global pest, the desert locust, shows two extreme phenotypes; a solitarious phase that is relatively harmless and a gregarious phase that forms swarms and causes extensive agricultural and economic damage. In the field, environmental conditions can drive isolated animals into crowded populations and previous studies have identified the biogenic amine serotonin as a key determinant of this transition. Here we take an integrated approach to investigate the neurochemical, physiological, and behavioral correlates defined by a laboratory based paradigm that mimics facets of swarm break down as gregarious locusts become isolated. Following isolation there was an increased propensity of locusts to avoid conspecifics, and show a reduced locomotion. Changes in choice behavior occurred within 1 h of isolation although isolation-related changes progressed with increased isolation time. Isolation was accompanied by changes in the levels of the biogenic amines dopamine, octopamine, and serotonin within the CNS within 1 h. Dopamine levels were higher in isolated animals and we focused on the role played by this transmitter in synaptic changes that may underpin solitarization. Dopamine reduced synaptic efficacy at a key central synapse between campaniform sensilla (CS) and a fast extensor tibiae motor neuron that is involved in limb movement. We also show that dopamine injection into the haemocoel was sufficient to induce solitarious-like behavior in otherwise gregarious locusts. Further, injection of a dopamine antagonist, fluphenazine, into isolated locusts induced gregarious-like behavior. This highlights that dopaminergic modulation plays an important role in the plasticity underpinning phase transition and sets a context to deepen the understanding of the complementary role that distinct neuromodulators play in polyphenism in locusts.

## Introduction

The phenotype of an animal can be influenced and modified by cues in the external environment. These allow animals to adapt to changes in their environment and may represent a fundamental component of evolutionary change (Thompson, 1991). Plasticity can result from changes in many factors, including temperature, diet, parasitism and population density, and can be reversible within the lifespan of an animal. Recently there has been intense interest in phenotypic plasticity in the light of the impact of climate change and its potential effects on populations of plants and animals and in ecological and evolutionary processes (see for example, Charmantier et al., 2008).

Phenotypic plasticity involves a variety of morphological, developmental, physiological and behavioral adaptations (Price et al., 2003; Pigliucci et al., 2006) and is found in both vertebrates and invertebrates. Among the invertebrates, insects represent an intensively studied group of animals showing phenotypic plasticity. Some aphids, for example, show density-dependent change from asexual to sexual reproduction depending on population size (Srinivasa and Brisson, 2012). Several species of grasshoppers, the swarm forming locusts, show some of the most striking examples of phenotypic plasticity (Pener and Yerushalmi, 1998; Cullen et al., 2010). The economically important pest species the desert locust, *Schistocerca gregaria*, expresses an extreme form of density-dependent polyphenism, or phase change (Opstad et al., 2004; Pener and Simpson, 2009). Locusts occur in two forms that differ in their morphological, physiological and behavioral characteristics representing a gregarious or swarming phase and a solitarious, non-swarming phase. At low population densities locusts avoid contact with conspecifics, are cryptic in appearance, fly mainly at night and do not swarm (Uvarov, 1977). They are green or brown as nymphs and pale yellow when mature. On the other hand, locusts in the gregarious phase actively group together, are yellow and black as nymphs, but bright yellow as adults (Uvarov, 1977). In this phase the locusts are highly active and fly during the day and can cause immense agricultural and economic damage by their voracious feeding on crops (Bullen, 1966).

Recent studies have used the experimentally tractable desert locust, *S. gregaria*, to unpick the physiological, molecular, and genetic mechanisms that underlie this polyphenism and the change in phase from the solitarious to the gregarious form (Claeys et al., 2006; Tanaka, 2006; Rogers et al., 2007; Geva et al., 2010; Lucas et al., 2010; Ott et al., 2012), as a greater understanding of phase change could lead to the development of new methods of pest control. Rogers et al. (2004) defined important changes in key transmitters and their precursors as a platform to defining the neurochemical mediators of phase change, and highlighted an important role for biogenic amines in polyphenism. Subsequently, it was shown that increased levels of serotonin in the CNS are both necessary and sufficient to drive the transition from the solitarious to the gregarious phase (Anstey et al., 2009). Further evidence for a key role for this transmitter in mediating the solitary to gregarious transition was supported by studies in which antagonists of serotonergic transmission were shown to inhibit phase change. This work was extended in the related migratory locust, *Locusta migratoria*, using a genome wide screen, and RNA interference, combined with pharmacological analysis to identify a role for dopamine in the same transition (Ma et al., 2011; Yang et al., 2014). Guo et al. (2011) showed that dopamine alone was not sufficient to complete transition, which instead required a more complex interplay between amines, particularly serotonin and dopamine. These results highlight the issue as to whether the two species differ in the neuromodulatory mechanisms underlying the transition from the solitarious to the gregarious phase. Simpson et al. (2011) point out that there is an increase in dopamine in the transition from the solitarious to gregarious phase (Rogers et al., 2004) in the desert locust but whose longer time course may not match the rapid transition in behavior. The inference from these studies on different species is that different mechanisms may well underpin the same transition in different species (Rogers et al., 2014).

Although the initial neurochemical studies focused on the solitarious to gregarious transition locusts also express a reverse transition from the gregarious to the solitarious phase, which again is associated with changes in the relative expression of biogenic amines and neurotransmitters (Rogers et al., 2004). However, we know little of the mechanism underlying this phase reversal as pointed out recently by Rogers et al. (2014). Is it simply a reduction in the amines known to generate plasticity or does it involve a change in other neurochemicals. How do the neurochemicals mediate neural and behavioral changes?

Here we use a simple measure of locust behavioral state following husbandry designed to generate gregarious and isolated states. We then use these animals to identify changes in the organism's neurochemistry, which highlights a possible role for the biogenic amine dopamine in phase reversal. We underpin the key determinants of function that control the neural networks that allow for the expression of distinct behaviors associated with the different phases. This enabled us to superimpose artificial changes in dopamine levels that support the notion that it causes physiological changes at the neural network level that modify behavior. We then draw together and discuss previously published pharmacological studies that establishes dopamine as a key neurochemical in phase reversal that generates a suite of physiological changes that impact on walking, jumping and flying. Finally we provide evidence to support the notion that different closely related species utilize different biochemical mechanisms to underpin the same phenotype.

## Materials and methods

### Locust husbandry

Experiments were carried out using adult desert locusts, *S. gregaria*, (Forskål) taken from crowded and solitarious colonies maintained at the University of Southampton. Gregarious locusts were reared in a controlled environment room in metal cages (39 × 39 × 45 cm) at a density of approximately 50–100 locusts per cage, and were fed daily on seedling wheat and dry oats. For experiments, 10 four-day-old adult locusts, post final molt, were moved to a small cage (20 × 20 × 20 cm) to ensure continued crowded conditions. These locusts were housed within the same gregarious colony room at a temperature 30 ± 1°C under a 12D: 12L h (Dark: Light) cycle, and fed daily with fresh seedling wheat and oats.

In a separate controlled environment room, maintained at a temperature 30 ± 1°C under a 12D: 12L (Dark: Light) cycle, 10 four-day-old adult gregarious locusts taken from the crowded colony were isolated in a way to prevent physical, visual and olfactory cues between locusts. Individual locusts were placed in plastic cages (20 × 20 × 20 cm), which were surrounded with patterned paper to prevent visual cues from other locusts and to provide each insect with a salient environment. Small holes were drilled in the bottom of the cage and a small hole (7 mm diameter) was drilled at the top of the cage. Plastic tubes (8 mm outside diameter × 1 mm thickness) were inserted into the top hole via Portex connectors. Tubes were fed to a Perspex air collection box from all cages and the air drawn from the room through the holes in the bottoms of the cages and out through the tube at the top and expelled from the colony using two air pumps (Air Cadet. 7530-65, Cole-Parmer Instrument Co., UK) housed outside the colony room. Airflow rate was measured using a glass flow meter (NG Series, CT Platon, France) at 126 ± 6.7 cm^3^/min.

### Behavioral assay

An assay was designed to study the behavioral responses of individual gregarious or isolated locusts based on a number of measures described by Roessingh et al. (1993), including behavioral choice, walking duration, distance, mean and maximum velocity. Experiments were performed in a rectangular glass arena (60 × 30 × 38 cm). The walls and floor were covered with tissue paper that was replaced after each experiment, and the outside covered in card to reduce visual stimulation. Opposite ends of the arena held a wire mesh cage that was either empty or contained a group of gregarious locusts (12 locusts). The end of the arena at which the stimulus cage was placed was changed randomly between each trial. A test locust was placed in a small release chamber (22 mm external diameter × 88 mm length) positioned at the midpoint along one wall of the arena. The release chamber was replaced after each experiment to prevent any potential confounding effects. The behavior of a test locust was recorded from above using a Sony DCR-DVD110E (Japan) digital video camera. Video recording started just before the test locust was placed in the release tube and stopped after the test locust reached either end of the arena, or after 10 min. Each locust was tested only once and all videos saved on a computer for subsequent frame-by-frame analysis.

### Electrophysiological recordings

A locust was placed ventral-side-up in a cleft in a modeling clay platform. The legs were fixed using more clay, leaving the tibia of the right hind leg free. The cuticle of the ventral thorax and underlying air sacs were removed. The metathoracic ganglion was exposed and supported on a wax-coated silver platform and restrained with small pins to isolate it from the movements of the thorax. The posterior connectives of the metathoracic ganglion were cut while the anterior connectives and lateral nerves remained intact. During the dissection and recording process, the thoracic cavity was continuously flushed with locust saline. A pair of 50 μm copper wires, insulated except at the tips, was inserted through small holes in the femur into the extensor muscle of a hind leg to stimulate it, and to evoke antidromic spikes in the fast extensor tibia motor neuron (FETi). Microelectrodes were made from borosilicate glass capillaries (1 × 0.58 mm interior diameter) pulled on a pipette puller (Kopf, Model 730 Needle Pipette Puller) and filled with 3 M potassium acetate, and with DC resistances of 50–80 MΩ. Before recording, the sheath of the ganglion was treated directly with protease (Sigma type XIV) for 1 min. A microelectrode was then inserted through the sheath and into the cell body of FETi. Intracellular recordings were made with the use of an Axoclamp 2A amplifier (Axon Instruments, USA) (Parker and Newland, 1995). To record the activity of campaniform sensilla (CS) a small piece of cuticle from the anterior face of the femur was removed to expose nerve 5B1 (N5B1) that contains the axon of the sensory neuron from the posterior CS. The nerve was placed on a pair of silver hook electrodes that were then isolated using petroleum jelly and spikes in the CS recorded extracellularly from N5B1 in the femur. All data were recorded on a computer using a Cambridge Electronic Design (Cambridge, UK) micro1401 A/D interface and Spike 2 (Version 7, Cambridge Electronic Design) software.

### Measurement of biogenic amines in the CNS

Individual locusts were quickly frozen using liquid nitrogen and the brain and metathoracic ganglion dissected out in cooled standard saline. Each ganglion was then placed in a micro glass homogenizer and homogenized in 50 μl of ice-cold 0.1 M perchloric acid containing 5 ng of 3, 4-dihydroxybenzylamine (DHBA) as an internal standard. After centrifugation of the homogenate (0°C, 15,000 rpm, 30 min), 40 μl of supernatant was collected. Biogenic amines in the sample were measured using high-performance liquid chromatography (HPLC) with electrochemical detection (ECD). The HPLC-ECD system was composed of a pump (EP-300, EICOM Co., Kyoto, Japan), an auto-sample injector (M-504, EICOM Co., Kyoto, Japan) and a C18 reversed-phase column (250 × 4.6 mm internal diameter, 5 μm average particle size, CAPCELL PAK C18MG, Shiseido, Tokyo, Japan) heated to 30°C in the column oven. A glass carbon electrode (WE-GC, EICOM Co.) was used for electrochemical detection (ECD-100, EICOM Co.). The detector potential was set at 870 mV vs. an Ag/AgCl reference electrode, which was also maintained at 30°C in a column oven. The mobile phase containing 0.18 M chloroacetic acid and 16 μM disodium EDTA was adjusted to pH 3.6 with NaOH. Sodium-1-octanesulfonate at 1.85 mM as an ion-pair reagent and CH_3_CN at 8.40% (v/v) as an organic modifier were added to the mobile phase solution. The flow rate was maintained at 0.7 ml/min. The chromatographs were acquired using the computer program PowerChrom (eDAQ Pty Ltd, Denistone East, NSW, Australia). The supernatants of samples were injected directly onto the HPLC column. After the acquisition, they were processed to obtain the level of biogenic amines in the same sample by the ratio of the peak area of substances to the internal standard DHBA. We used a standard mixture for quantitative determination that contained amines, precursors and metabolites. Twenty compounds at 100 ng/ml each were DL-3, 4-Dihydroxy mandelic acid (DOMA), L-β-3,4-Dihydroxyphenylalanine (DOPA), L-Tyrosine (Tyr), N-acetyloctopamine (Nac-OA), (-)-noradrenaline (NA), 5-Hydroxy-L-tryptophan (5-HTP), (-)-adrenaline (A), DL-Octopamine (OA), 3,4-Dihydroxybenzylamine (DHBA, as an internal standard), 3,4-Dihydroxy phenylacetic acid (DOPAC), N-acetyldopamine (Nac-DA), 3,4-Dihydroxyphenethylamine (DA), 5-Hydroxyindole-3-acetic acid (5-HIAA), N-acetyltyramine (Nac-TA), N-Acetyl-5-hydroxytryptamine (Nac-5HT), Tyramine (TA), L-Tryptophan (Trp), 3-Methoxytyramine (3-MTA), 5-Hydroxytryptamine (5-HT), 6-Hydroxymelatonin (6-HM). Nac-OA Nac-DA and Nac-TA were synthesized by Dr. Matsuo (Keio University, Japan). All other substances were purchased from Sigma-Aldrich.

### Cuticle stiffness

To measure cuticle stiffness the tibia was cut from the hind leg and fixed dorsal-side-up on steel holders using cyanoacrylate glue. Two weights (20 and 30 g) were hung sequentially from the middle of the tibia using cotton thread and the resulting bending distances of the tibia determined from digital images. The Beam bending equation was used to calculate the bending stiffness (*EL*) of the tibia:

$$EL=F\times L^{3}/48\times B$$

where *F* is the weight used, *L* is the length of the tibia between the holders, *B* is the tibial displacement and 48 is a constant.

### Kick force measurement

A S100SMD (SMD, USA) thin film load cell was placed in contact with the tibia and was used to measure the kick force. A pair of 50 μm copper wires, insulated except at the tips, was inserted through small holes into the extensor muscle of a hind leg to stimulate the muscle and evoke a kick. All recordings were written directly to a computer with a CED 1401 A/D converter and displayed using Spike 2 software.

### Pharmacological analyses

For pharmacological analysis dopamine hydrochloride and two dopamine receptor antagonist, chlorpromazine hydrochloride and fluphenazine dihydrochloride, were obtained from Sigma-Aldrich, (UK) and dissolved to final concentrations in standard locust saline. Dopamine was tested at concentrations of 1, 5, and 10 mM, chlorpromazine at 0.1, 1, and 5 mM, and fluphenazine at 100 μM, 500 μM, and 5 mM at concentrations used in previous studies (Unoki et al., 2005; Ma et al., 2011).

### Injection of dopamine and its antagonists

Dopamine hydrochloride (Sigma-Aldrich, UK) was dissolved in standard locust saline to make a 422 mM solution. 4 μl of this solution was injected directly into the thoracic haemocoel of each locust via the ventral abdomen between the second and third abdominal segments using a microsyringe (Sigma-Aldrich, UK), following drug concentration, volumes, and methods published elsewhere (Ma et al., 2011). Each locust was restrained ventral surface uppermost in Plasticine©, and the tip of the syringe needle maneuvered into the thoracic haemocoel. Ten gregarious locusts were used for each experiment and each experiment replicated six times. Five of these gregarious locusts were injected with 4 μl of 422 mM dopamine solution (treatment animals) whereas the remaining five received 4 μl of standard saline (control animals). Following injection locusts were returned to the crowded colony for 1 h before their behavior was assayed. Solitarious locusts were also treated in a similar manner, with 10 solitarious locusts used for each experiment. Five of these locusts were injected with 4 μl of 422 mM dopamine as a treatment group, and five locusts injected with 4 μl of standard saline as controls (Ma et al., 2011). Following injections locusts were housed individually under isolated conditions for 1 h before their behavior was assayed.

Two different dopamine receptor antagonists, chlorpromazine (CPZ) (see Supplementary Data, Figure E) and fluphenazine were used to block the action of dopamine. These D1 and D2 receptor antagonists were chosen as they have been used in other studies in invertebrates (Degen et al., 2000; Unoki et al., 2005; Mustard et al., 2010). Chlorpromazine hydrochloride (Sigma-Aldrich, UK) was dissolved in standard locust saline to make a 56 mM solution, and 3 μl of this solution was injected directly into the thoracic haemocoel using the same methods described above. The concentrations and volumes chosen were based on previous studies (Ma et al., 2011). Fluphenazine dihydrochloride (Sigma-Aldrich, UK) was dissolved in standard locust saline to make a 500 μM solution and 5 μl of this solution was injected directly into the thoracic haemocoel using the same methods described above. This concentration was chosen based on previous studies (Ma et al., 2011).

### Statistical analysis

Data from experiments on behavioral choice were analyzed using a Chi-squared test. Analysis of variance was carried out on normally distributed data using ANOVA and Students *T*-tests, whereas non-normally distributed data was analyzed using Kruskal–Wallis and Mann–Whitney *U*-tests.

## Results

### Isolation leads to changes in behavioral choice

**Figure 1:**
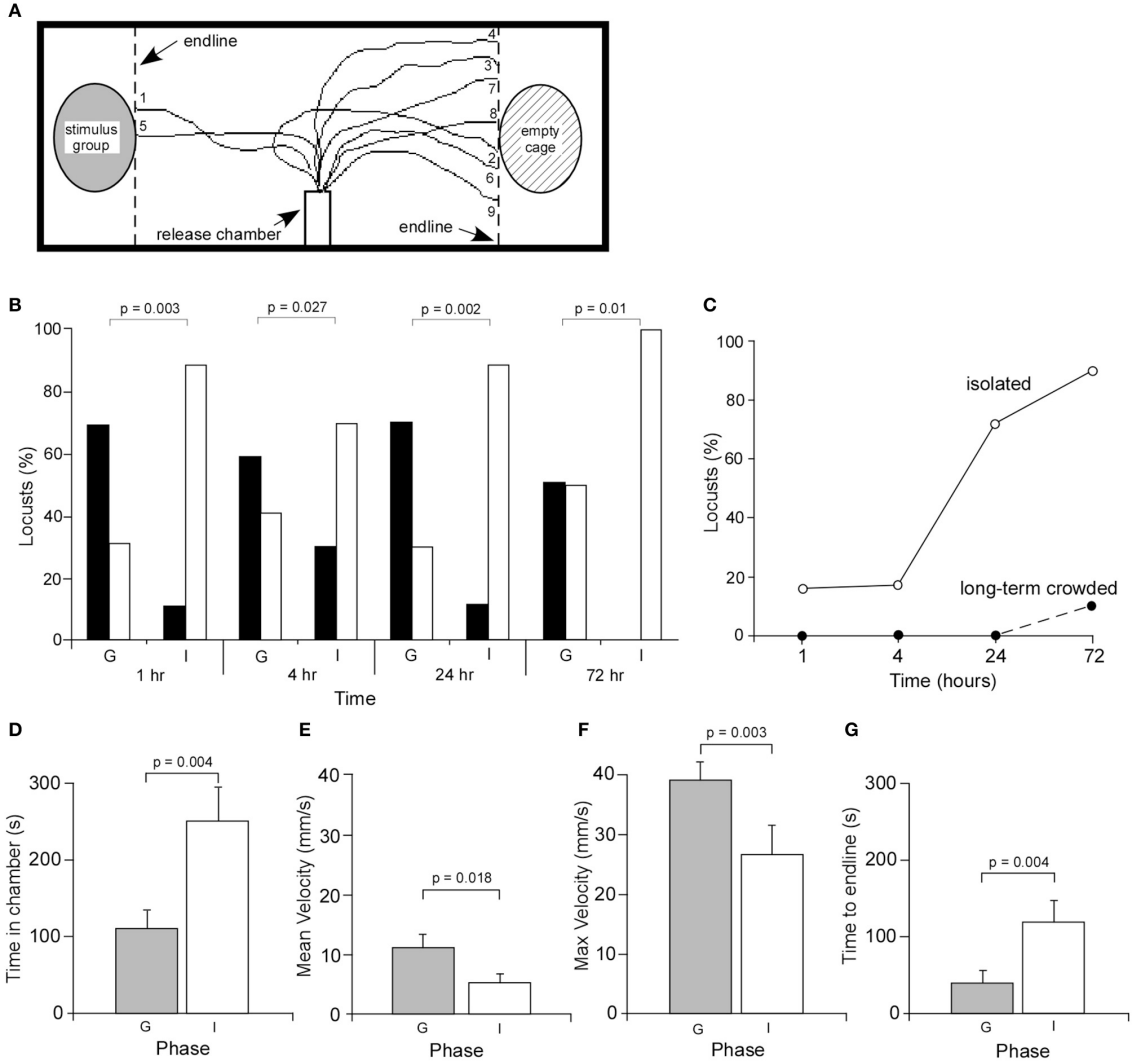
**Behavioral responses of gregarious and isolated locusts. (A)** Experimental setup showing the release chamber and the walking tracks of nine isolated locusts. **(B)** Behavioral choices of age-matched gregarious (control) (G) and isolated (I) locusts. Locust avoiding the stimulus group and remaining within the release chamber were summed and together used as a measure of overall avoidance. Black bars indicate attraction, white bars indicate avoidance. Probability values were based on Chi-squared statistical tests. **(C)** Graph showing the percentage of animals remaining within the release chamber for isolated and age-matched crowded animals. **(D)** The mean time (±s.e.m.) a locust spent in the release chamber was significantly greater in isolated locusts, excluding those that did not emerge within the 10 min duration of the analysis. The mean **(E)** and maximum **(F)** walking velocities of gregarious locusts were significantly greater than isolated locusts. **(G)** The time for locusts to cross the test arena to the end lines was significantly longer in isolated locusts.

Experimentally induced phase change can be assayed by measuring locomotion and, or how, it is used to express a preference or avoidance of conspecific animals (Hägele and Simpson, 2000; Tanaka and Nishide, 2013). Based on this we developed an experimental protocol in which animals were raised in a long-term crowded culture before being subjected as adults, in cohorts, to isolation in cages in which gregarising mechanical, olfactory, and visual cues were removed. This was done by placing long-term gregarious animals in isolation chambers for increasing periods of time. To determine the effects of isolation on the choice behavior of otherwise long-term gregarious locusts, the individuals were placed in a release chamber at the midline of a test arena which had an empty cage or one containing 12 long-term gregarious at opposite ends. Locusts were allowed to move freely and their behavior recorded by video for a maximum of 10 min or until they reached the endline at either end of the test arena (Figure 1A). The walking tracks of the locusts were then plotted and analyzed to quantify walking kinematics (Figure 1A). The time course of behavioral change was investigated by placing long-term gregarious locusts in isolation for periods of 1–72 h and age-matched long-term crowded locusts tested as controls for each time period of isolation.

After 1 h of isolation, an analysis of the behavior of 19 locusts showed that 2 were attracted to the stimulus group of locusts at the end of the test arena, 14 avoided the stimulus group, while three remained in the release tube (Figures 1B,C). By contrast, 11 of 16 age-matched long-term gregarious locusts were attracted to the stimulus group, while only five locusts avoided the stimulus group. None stayed in the release chamber. Statistical analysis showed that there was a significant difference between the behavior of gregarious and isolated locusts [χ^2^_(1)_ = 12.61, *P* = 0.0003, *n* = 16 gregarious, *n* = 19 isolated]. Following isolation for 4 h, 9 of 30 locusts were attracted to the stimulus group, 16 avoided the stimulus group, while 5 remained in the release chamber. By comparison, 17 of 29 age-matched gregarious locusts were attracted to the stimulus group, while 12 avoided the stimulus group. Statistical analysis again showed that there was a significant difference in the choice behavior between the two groups after 4h in isolation [χ^2^_(1)_ = 4.9, *P* = 0.027, *n* = 29 gregarious, *n* = 30 isolated] (Figures 1B,C). After 24 h of isolation, 3 of 18 locusts avoided the stimulus group, two were attracted to the stimulus group while 13 remained in the release chamber. Fourteen of 20 age-matched gregarious locusts were attracted to the stimulus group while six avoided the stimulus group. There was again a significant difference in choice behavior between the two groups [χ^2^_(1)_ = 13.47, *P* = 0.0002, *n* = 20 gregarious locusts, and *n* = 18 isolated locusts]. Finally, following isolation for 72 h 1 of 10 isolated locusts avoided the stimulus group while 9 remained in the release chamber. By contrast, 5 of 10 age-matched locusts were attracted to the stimulus group, four avoided the stimulus group and one remained in the release chamber. Statistical analysis again showed that there was a significant difference between behavioral choices of the two groups [χ^2^_(1)_ = 6.66, *P* = 0.01, *n* = 10 gregarious, *n* = 10 isolated]. The greater the time in isolation the greater the number of locusts remaining within the release chamber for the 10 min test period (Figure 1C). These changes in behavior were completely reversible across the measured time course by returning locusts to crowded conditions (Supplementary Data, Figure A).

These results demonstrate that isolated locusts show distinct differences in behavior within 1 h of isolation compared to long-term crowded locusts, by remaining in the release chamber before making their choice, and once having made a choice show a clear preference to avoid conspecifics.

### Isolation results in changes in walking behavior

The effect of isolation on walking behavior was determined from a frame-by-frame analysis of video recordings. Five parameters were analyzed, including the time to leave the release chamber, the mean and maximum velocities, the distance traveled from the release tube to the end line/stimulus cage and the time to cross the arena to the end line. Of the locusts isolated for 72 h that left the release chamber within the 10 min experimental period, those animals took significantly longer to leave the chamber (252 ± 42 s, mean ± Standard Error of the Mean, SEM) compared to gregarious locusts (111.7 ± 20 s, mean ± s.e.m.) (Students *T*-test, *P* = 0.005, *n* = 13 gregarious and *n* = 11 isolated) (Figure 1D).

The walking behavior of gregarious locusts was then compared to that of locusts isolated for 72 h. Results showed that both groups covered approximately the same distance from the release chamber to the stimulus cage suggesting there was no significant difference in the sinuosity of their walking paths, with gregarious locusts covering 211 ± 10 mm (mean ± s.e.m.) and isolated locusts covering 234 ± 20 mm (Students *T*-test, *P* = 0.58, *n* = 13 gregarious and *n* = 11 isolated locusts). Analysis of walking showed that gregarious locusts had significantly higher mean (Figure 1E) and maximum (Figure 1F) walking velocities of 11.5 ± 1 mm/s (Figure 1E) and 39.5 ± 3 mm/s, respectively, compared to isolated locusts with mean (Figure 1E) and maximum (Figure 1F) velocities of 5.4 ± 1 mm/s and 26.9 ± 5 mm/s, respectively (Students *T*-test, *P* = 0.018, *n* = 13 gregarious and *n* = 11 for isolated locusts for mean velocity; *P* = 0.027, *n* = 13 gregarious and *n* = 11 isolated locusts for maximum velocity). These differences in walking velocity reflect the times the two groups took to cross the arena with gregarious locusts reaching the end line in significantly shorter times of 42.5 ± 11.2 s compared to 120.8 ± 28 s in isolated animals (Figure 1G) (Students *T*-test, *P* = 0.004, *n* = 13 gregarious and *n* = 11 isolated locusts.

These results clearly demonstrate that isolation leads to distinct changes in walking movements.

### Biochemical analysis of the metathoracic ganglion

The movements of the legs of locusts are produced and controlled by neural networks within each hemiganglion of the thorax (i.e., the prothoracic leg by the prothoracic ganglion, the mesothoracic leg by the mesothoracic ganglion, and the metathoracic (hind) leg by the metathoracic ganglion). Given the importance of the hind leg, and exteroceptors on it, in initiating gregarisation (Hägele and Simpson, 2000) we analyzed the levels of four key amines in individual metathoracic ganglia of animals isolated for periods of 1–72 h.

**Figure 2:**
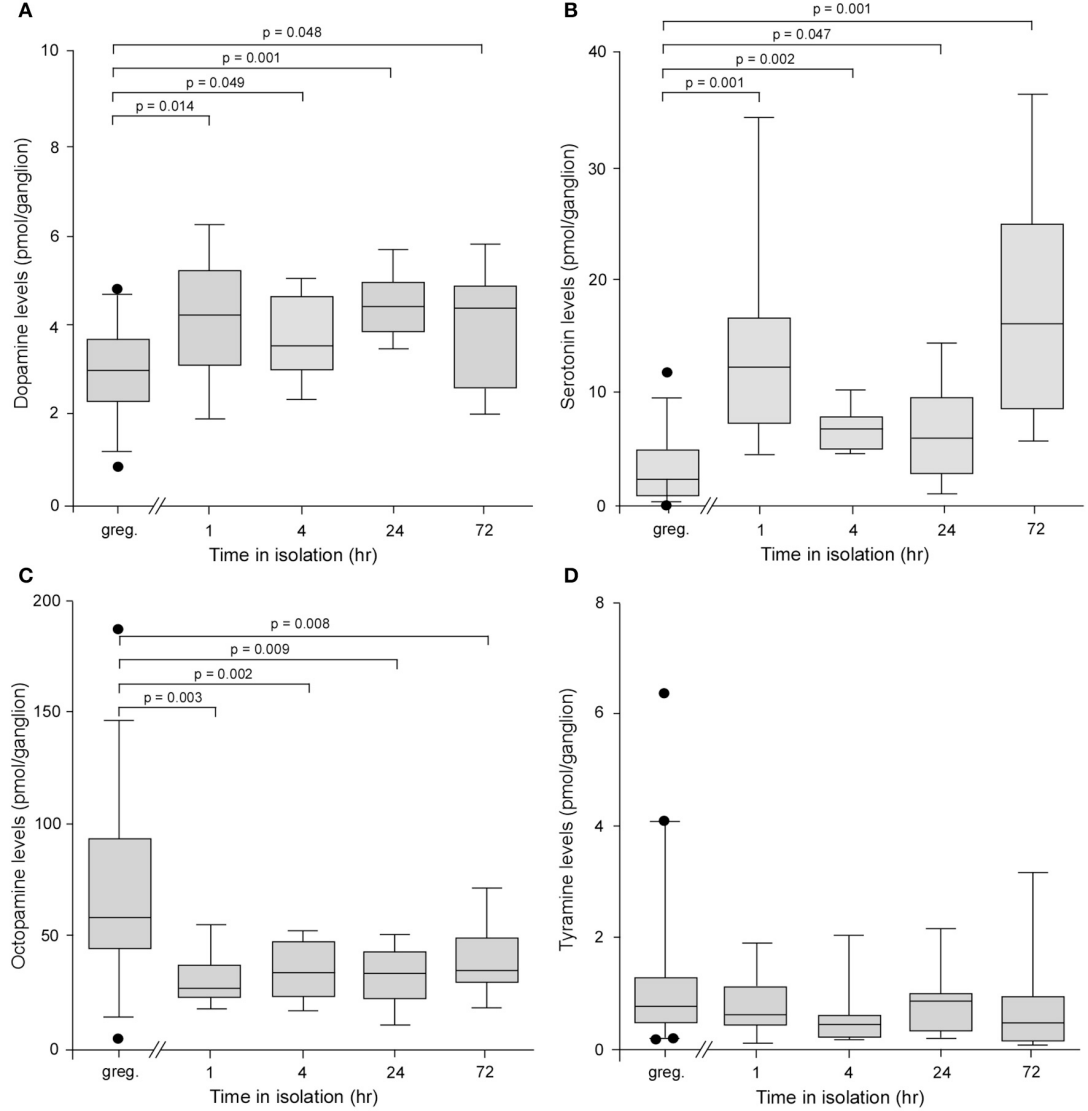
**Analysis of the levels of four biogenic amines isolated from individual metathoracic ganglia using HPLC with electrochemical detection. (A)** Box and whisker plots showing median, interquartile range, and minimum and maximum values of the levels of dopamine in the metathoracic ganglion that increased significantly in locusts after only 1 h in isolation compared to gregarious controls. **(B)** Serotonin levels increased significantly in the metathoracic ganglion after 1 h in isolation. **(C)** By contrast the levels of octopamine decreased significantly after 1 h in isolation. **(D)** The levels of tyramine showed no difference between gregarious and isolated locusts. Results are based on *n* = 9–11 animals tested at each time point for isolated and long-term gregarious groups and significance tested using Kruskal–Wallis, followed by pair-wise Mann–Whitney *U*-tests, indicated above each graph.

The levels of dopamine in individual ganglia were significantly higher in locusts isolated for as little as 1 h compared to long-term crowded locusts, and this elevation maintained for periods up to 72 h, the longest time point we analyzed (Kruskal–Wallis Test, *H* = 17.91, d.f. = 4, *P* = 0.0013)(Figure 2A). Pair-wise Mann–Whitney *U*-test comparisons showed that at all time points in isolation dopamine levels were higher than in control gregarious animals (Mann–Whitney *U*-test probabilities given on Figure 2). For example, gregarious locusts expressed 3.0 ± 0.16 pmol/ganglion, whereas locusts isolated for as little as 1 h had significantly higher levels of 4.19 ± 0.45 pmols/ganglion (Mann–Whitney *U*-test, *P* = 0.0141). Interestingly, the levels of serotonin (5-HT) were also significantly higher in locusts isolated for as little as 1 h and, as for dopamine, this elevation in levels was maintained for up to 72 h in isolation (Kruskal–Wallis Test, *H* = 35.48, d.f. = 4, *P* = 0.0001) (Figure 2B). Pair-wise Mann–Whitney *U*-test comparisons again showed that at all time points in isolation serotonin levels were higher than in control gregarious animals (Mann–Whitney *U*-test probabilities given on Figure 2B). The changes in these two biogenic amines were selective as the levels of a different amine, octopamine, were reduced in isolated animals compared to gregarious locusts across the 72 h time course investigated (Kruskal–Wallis test, *H* = 26.21, d.f. = 4, *P* = 0.0001) (Figure 2C). Pair-wise Mann–Whitney *U*-test comparisons showed that at all time points in isolation octopamine levels were lower than in control gregarious animals (Mann–Whitney *U*-test probabilities given on Figure 2C). Finally a fourth biogenic amine, tyramine, with key neuromodulatory functions across phyla, showed no change in the low level of expression between long-term crowded and isolated locusts (Kruskal–Wallis test, *H* = 8.74, d.f. = 4, *P* = 0.068) (Figure 2D).

Changes in the absolute and relative levels of these four amines were also found in the brains of animals isolated for 24 h although in this neural structure the serotonin levels were reduced in the animals subjected to isolation (Supplementary Figure B).

These observations highlight specific overt changes in the biogenic content of neural tissue as the animal expresses behavioral phase change, and implicate dopamine, serotonin and octopamine as likely candidates in reorganizing the nerve signaling underpinning the gregarious to solitarious transition.

### Effects of isolation at an identified synapse

**Figure 3:**
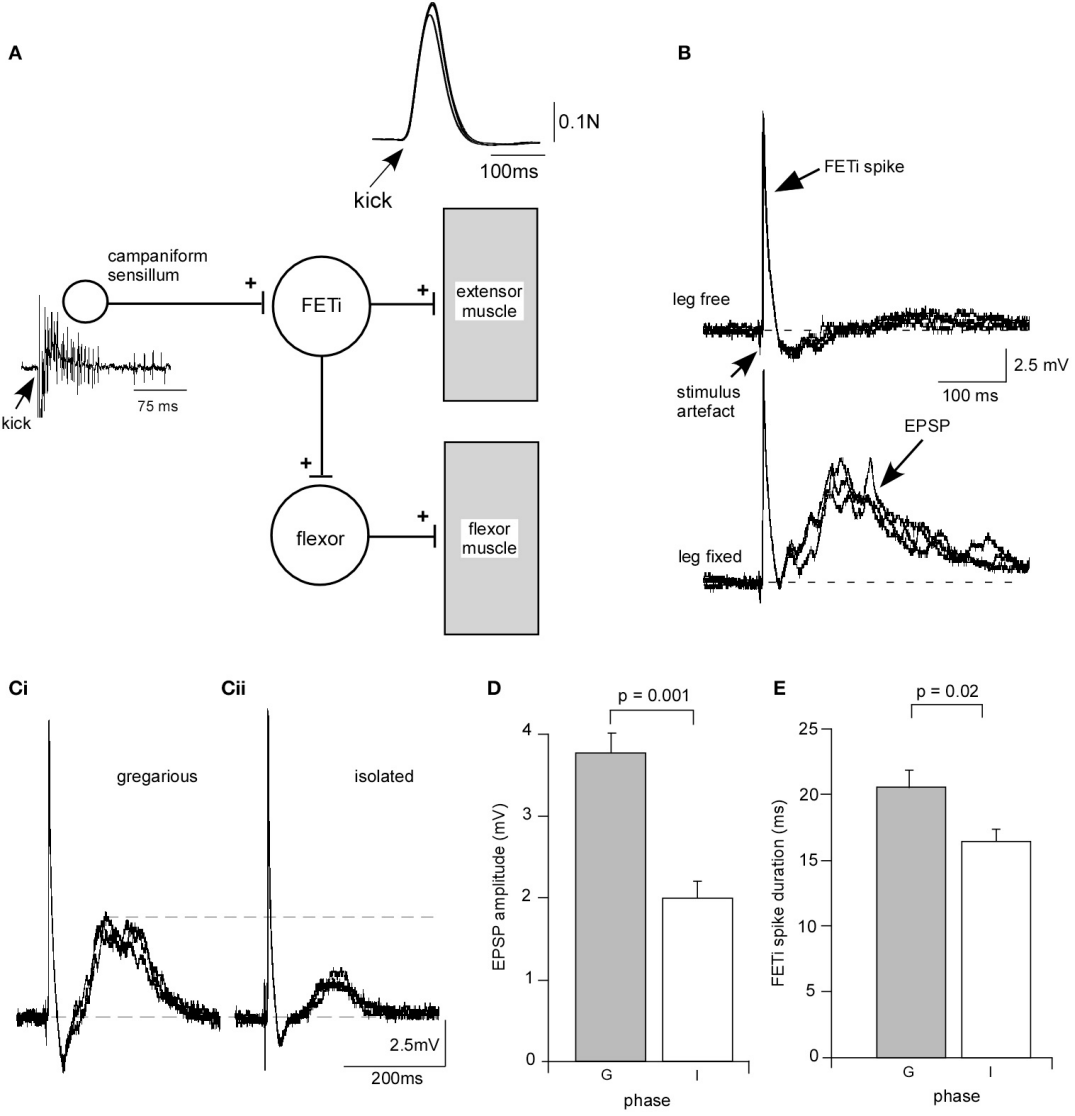
**Isolation leads to a change in synaptic strength at an identified synapse between campaniform sensilla and the fast extensor motor neuron (FETi). (A)** Diagram of the reflex loop. Electrical stimulation of the extensor muscle leads to a rapid extension of the tibia (kick) monitored by fixing the leg against a load cell. The kick activates campaniform sensilla on the leg that generate a burst of spikes and an antidromic spike in FETi. **(B)** Example recording from a gregarious locust showing that with the leg free to move an antidromic spike is recorded in the cell body of FETi. With the leg fixed the antidromic spike in FETi is followed by a compound EPSP generated by monosynaptic input from the afferents of the campaniform sensilla. **(C)** The compound EPSP in a locust isolated for 3 days is smaller than in a gregarious locust. **(D)** The mean EPSP amplitude in FETi was significantly smaller in locusts isolated for 3 days (I), and (**E**) the mean FETi spike duration was shorter in isolated locusts (I) than in gregarious controls (G). Students *T*-test, *P* < 0.05.

We analyzed the effect of isolation on a synapse between CS, mechanosensory receptors responsible for monitoring stress on the cuticle, and the fast extensor motor neuron (FETi) (Figure 3A). FETi is involved in jumping in the locust, another behavior that changes during phase transition (Uvarov, 1977) and also receives inputs from proprioceptors in the legs activated during walking in parallel with the slow extensor motor neuron (SETi), with similar dynamics and response properties (Newland and Kondoh, 1997). FETi also produces spikes in parallel with SETi during some limb movements (Page et al., 2008).

When FETi produces a spike it activates the extensor muscles to produce a kick or jump. During the kick/jump extensor muscle activation leads to bending forces on the cuticle that activate the CS that in turn produce positive feedback onto FETi in the form of a cholinergic compound monosynaptic excitatory postsynaptic potential (EPSP) (Parker and Newland, 1995) that follows the FETi spike at short latency when the leg was fixed (Figures 3A,B). We analyzed the effects of isolation for 72 h on this input to FETi. The amplitude of the EPSP mediated in fixed leg preparations was significantly reduced in locusts that had been isolated for 3 days. The mean amplitude of the compound potential of 3.77 ± 0.24 mV (mean ± s.e.m) (*n* = 12) in gregarious locusts was reduced to 1.98 ± 0.22 mV (*n* = 12) in isolated locusts (Figures 3C,D) (Students *T*-test, *P* = 0.001). While isolation had no effect on spike height it led to a significant reduction in spike duration (measured from the start of the spike to where it returned to the same voltage) following 72 h in isolation, from 32.92 ± 1.7 ms (*n* = 12) compared to 41.22 ± 2.7 ms in gregarious locusts (Students *T*-test, *P* = 0.02) (Figure 3E). Although we observed no clear or overt change in the morphology of cuticle it is clear that biomechanics are important to this sensory integration. Thus, we carried out a series of control experiments to determine whether isolation-induced plasticity at the CS-FETi synapse was due to changes in the stiffness of the cuticle affecting sensory input, the force output of a kick that could influence cuticle stress, the mass of the muscle, the length of the limb segments, or the spike frequency of the CS between crowded and isolated locusts (Supplementary Figure C). These control tests all proved to shown no differences between isolated and gregarious animals and taken together the results show that the solitarisation paradigm leads to significant changes at the synapse between identified neurons involved in controlling the movements of the hind leg, reducing the strength of synaptic input and reducing excitability.

### Pharmacological analysis of the effects of dopamine on the CS-FETi synapse

**Figure 4:**
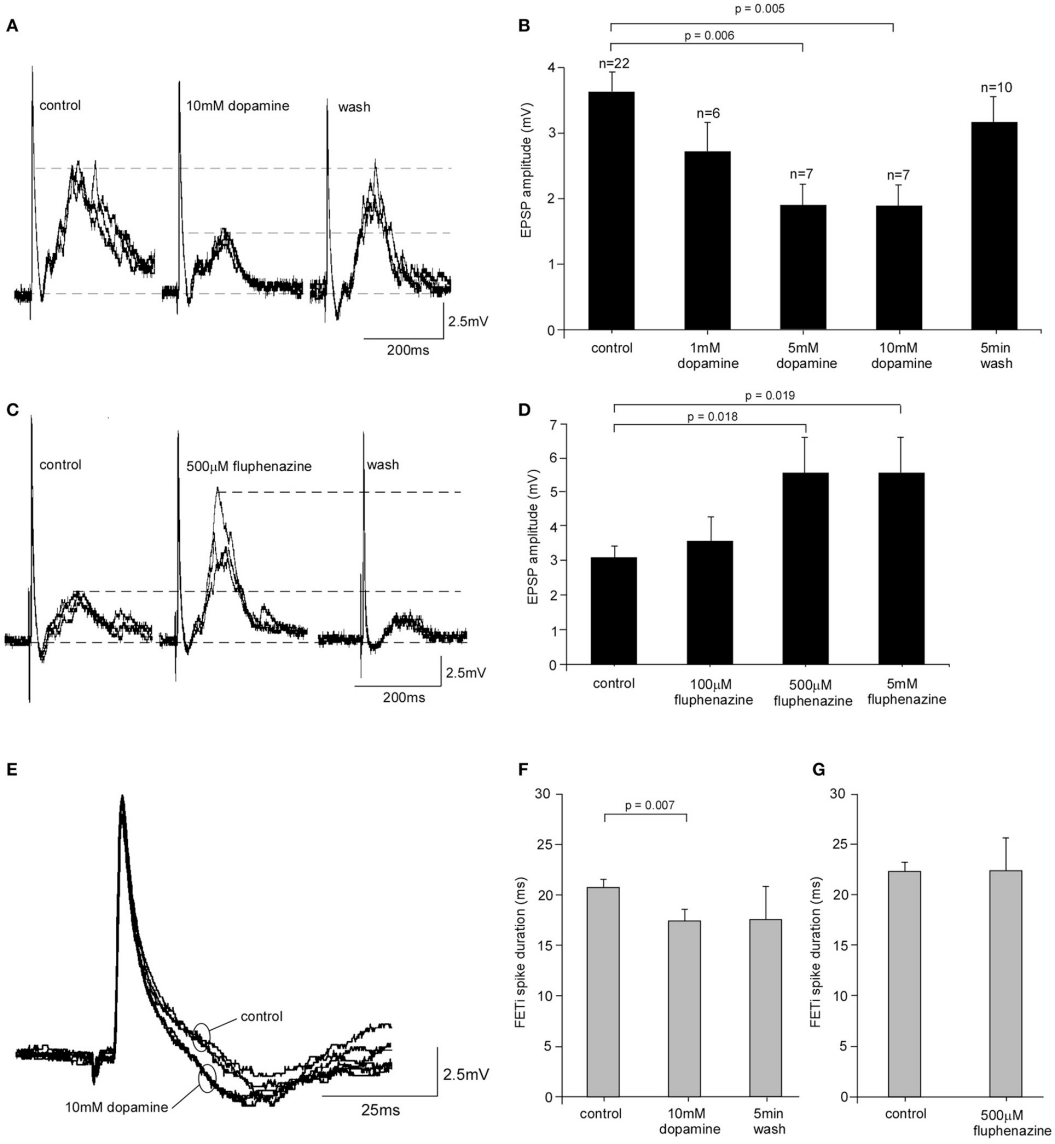
**Pharmacological analysis of FETi responses in gregarious locusts. (A)** 10 mM dopamine caused a reduction in the amplitude of the compound EPSP in FETi after 5 min bath application that reversed following a 5 min wash in normal saline. **(B)** The reduction in EPSP amplitude was concentration dependent (Students *T*-test, *P* < 0.05). **(C)** Bath application of the dopamine antagonist fluphenazine increased the amplitude of the compound EPSP in FETi after 5 min application, and the response returned to control levels after a 5 min wash. **(D)** The increase in EPSP amplitude caused by fluphenazine was concentration dependent (Students *T*-test, *P* < 0.05). **(E)** Superimposed sweeps from one locust shows that FETi spike duration is reduced during 10 mM dopamine application. **(F)** The reduction in spike duration was statistically significant (Students *T*-test, *P* < 0.05) and reversed after a 5 min wash. **(G)** Fluphenazine had no significant effect on the duration of the FETi spike.

Given the increased levels of dopamine found in the metathoracic ganglion in isolated locusts we investigated whether dopamine had the potential to modify the CS-FETi EPSP. We tested a range of dopamine concentrations (1, 5, and 10 mM) by superfusion over the ganglion *in vivo*. In gregarious locusts EPSP amplitude decreased significantly after 5 min perfusion of dopamine with increasing concentrations causing greater reductions in amplitude [One-Way ANOVA, *F*_(4, 47)_ = 4.40, *P* = 0.004] (Figures 4A,B). The effects of dopamine reversed following a 5 min wash period with normal saline. *Post hoc* Tukey tests showed that there were significant differences between the mean EPSP amplitudes at control, 5 and 10 mM dopamine concentrations (*P* = 0.006 and 0.005, respectively). Dopamine also significantly reduced the amplitude of the CS-FETi EPSP in locusts isolated for 72 h (Supplementary Figure D) suggesting that endogenous dopamine was not of a sufficient level to exert maximum EPSP changes.

We then tested the specificity of dopamine by analyzing the effects of dopamine antagonists on the CS-FETi EPSP. We first tested the effects of CPZ on the CS-FETi EPSP following its demonstrated effects on behavioral phase change in *L. migratoria* by the Kang group [15]. However, we found that CPZ had a deleterious effect on locusts, by rapidly abolishing neural activity, with many locusts dying, even at concentrations previously used elsewhere (Supplementary Figure E). We therefore tested the effect of a different dopamine antagonist, fluphenazine, on the amplitude of the CS-FETi EPSP (Figures 4C,D). The amplitude of the EPSP was dependent on fluphenazine concentration, with greater concentrations of the antagonist causing the largest amplitude EPSP [One-Way ANOVA, *F*_(3, 20)_ = 3.15, *P* = 0.034]. The co-application of dopamine and fluphenazine antagonized the increase in EPSP amplitude caused by dopamine alone (Supplementary Figure D).

We also tested the effects of dopamine and fluphenazine on FETi spike properties of gregarious locusts. Neither dopamine nor fluphenazine affected the FETi spike amplitude. Results showed, however, that 10 mM dopamine caused a significant decrease in the duration of the FETi spike after 5 min of application (Figures 4E,F) from 21.44 ± 0.38 ms in control gregarious locusts to 18.13 ± 1.00 ms following application of 10 mM dopamine (Students *T*-test, *t* = 3.09, *P* = 0.007, d.f. = 16). The effects of dopamine were reversed following a 5 min wash in normal saline. Further analysis of the effect of fluphenazine on FETi spike duration showed that 500 μM fluphenazine alone had no effect (Students *T*-test, *t* = 0.0039, *P* < 0.05, *n* = 6) (Figure 4G).

These results show that dopamine applied to the thoracic ganglia leads to changes in EPSP amplitude and spike duration in FETi, similar to those caused by isolation. The results we obtained using the dopaminergic antagonist suggest there is an intrinsic modulatory role for dopamine in the metathoracic ganglion.

### Effect of dopamine on behavioral choice

**Figure 5:**
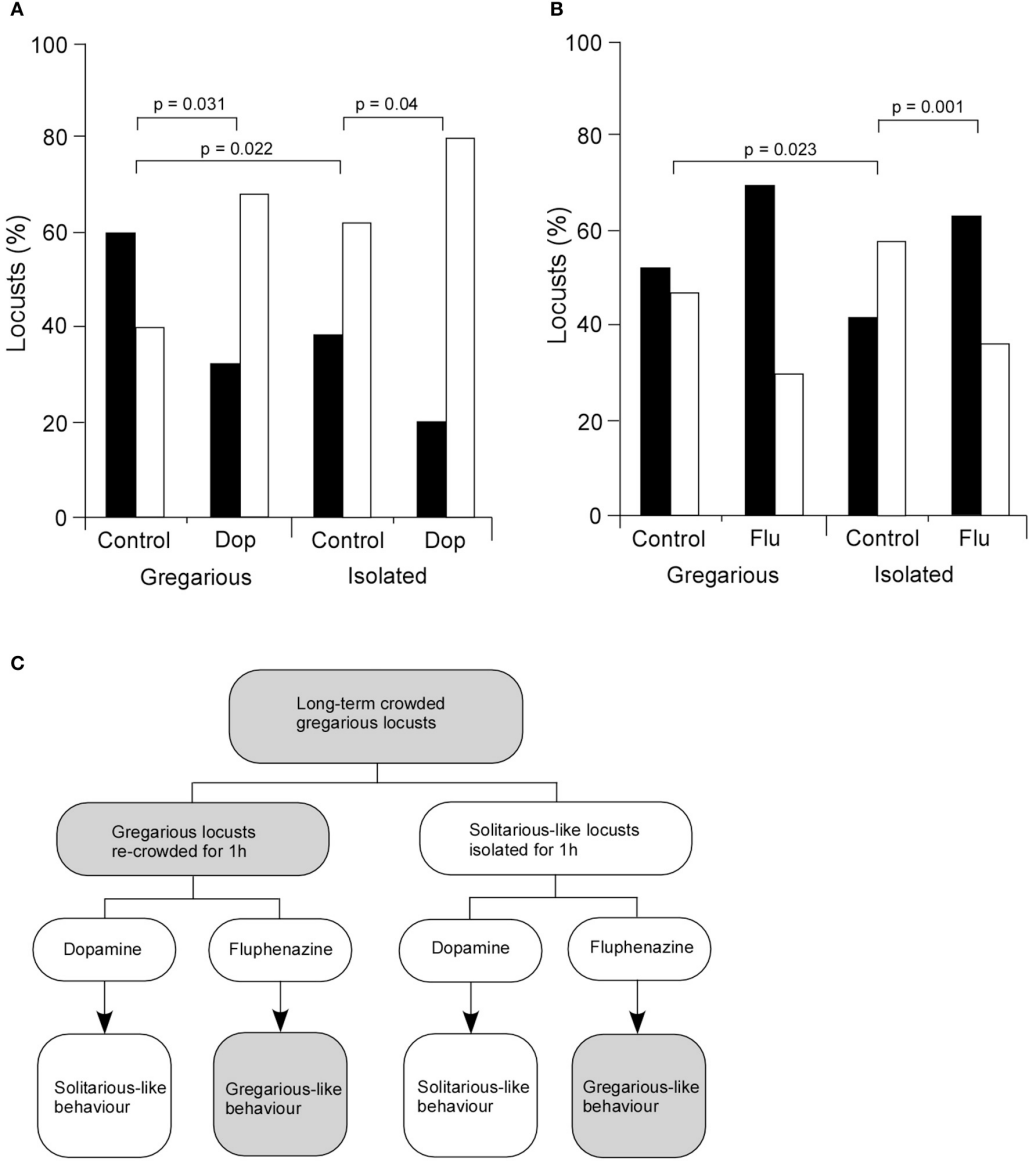
**Dopamine mediates changes in behavior. (A)** The injection of dopamine into the thoracic haemocoel of gregarious locusts causes a significant change in their behavior to show a preference to avoid the stimulus group in choice tests. Control locusts were injected with the same volume of standard locust ringer. Dopamine injected into isolated locusts enhances their normal preference to avoid other locusts. **(B)** By contrast, fluphenazine injected into gregarious locusts enhanced their preference to be attracted to the stimulus group, while it reversed the preferences of isolated locusts from avoidance to attraction to the stimulus group. Black bars indicate attraction, white bars indicate avoidance. **(C)** Summary of behavioral responses. Dopamine changes the normal behavior of gregarious locusts by generating solitarious-like avoidance behavior. Fluphenazine enhances the gregarious-like attraction behavior of gregarious locusts. By contrast dopamine enhances the avoidance behavior of isolated locusts, while fluphenazine reverses their choice preference to show attraction. Data in **(A)** and **(B)** were analyzed with Two-Way ANOVA followed by *post hoc* Tukey tests whose probabilities are shown about the graphs.

Given the increased level of dopamine in isolated animals and its marked effect on a central synapse *in vivo*, we then asked how dopamine affected the behavioral choices of long-term crowded locusts isolated for 3 days. Dopamine (4 μl of 422 mM dopamine) or saline (as a control) were directly injected into the thoracic haemocoel and locusts placed in isolated or crowded colonies for 1 h before experimentation. Results showed that for gregarious locusts 60% of control animals injected with saline were attracted to the stimulus group (33 of 55) while 40% avoided them (22 of 55) (Figures 5A,C). By contrast gregarious locusts injected with 4 μl of 422 mM dopamine showed a different behavior with only 32% (8 of 25) of locusts attracted to the stimulus group while 68% avoided them (17 of 25). Isolated locusts that underwent the same dopamine and saline treatments as described above were also tested and analysis of their behavior showed that 38% (21 of 55) of the control (saline injected) isolated locusts were attracted to the stimulus group and 62% (34 of 55) avoided them, while of the isolated locusts injected with dopamine 20% (5 of 25) were attracted to the stimulus group while 80% (20 of 25) avoided them (Figures 5A,C). Thus, for both gregarious and isolated locusts dopamine injection increased the numbers of locusts avoiding the stimulus group (solitarious-like behavior, Figure 5C). Statistical analysis showed that the difference in the mean values among the different levels of treatment was greater than would be expected by chance after allowing for effects of differences in phase. There was therefore a statistically significant interaction between locust phase and treatment [Two-Way ANOVA, *F*_(1, 28)_ = 0.004, *n* = 11 control groups, 5 dopamine groups (each group five locusts), *P* > 0.05]. *Post hoc* statistical analysis showed that there were significant differences in choice behavior between the control and dopamine treated gregarious locusts (*Post hoc* Tukey test, *P* = 0.031). Moreover, there was also a statistically significant difference between the control and dopamine treated isolated locusts (*Post hoc* Tukey test, *P* = 0.040), with statistically more dopamine treated animals avoiding the stimulus group [solitarious-like behavior (Figure 5C)]. Finally, there was a statistically significant difference between the control gregarious locusts and the control isolated locusts (*Post hoc* Tukey test, *P* = 0.022) (Figure 5B) as would be expected from their normal change in behavior due to isolation. These observations support a role for dopamine in mediating solitarious-like behavior in gregarious locusts, but not in the reverse transition (summarized in Figure 5C).

The corollary of this involved investigation of a dopamine antagonist, fluphenazine, that selectively blocked exogenous and endogenously mediated modulation of FETi (Figures 5B,C). Analysis of the choice behavior of control gregarious locusts (injected with 5 μl saline) showed that 53% (*n* = 29) were attracted to the stimulus group while 47% (*n* = 26) avoided them, whereas of fluphenazine (5 μl of 500 μM fluphenazine) treated gregarious locusts 70% (*n* = 21) were attracted to the stimulus group while 30% (*n* = 9) avoided them (Figure 5B). By contrast, analysis of the behavior of isolated locusts showed that of the saline-injected control locusts 42% (*n* = 23) were attracted to the stimulus group while 58% (*n* = 32) avoided them, whereas of isolated locusts injected with fluphenazine 63% (*n* = 19) were attracted to the stimulus group while 37% (*n* = 11) avoided them (Figure 5B). Statistical analysis showed that the difference in the mean values among the different levels of treatment was greater than would be expected by chance after allowing for the effects of differences in locust phase [Two-Way ANOVA, *F*_(1, 30)_ = 0.006, *n* = 11 control groups, six fluphenazine groups (each group five locusts), *P* < 0.05]. *Post-hoc* analysis showed there was no significant difference between the control and the treatment animals within gregarious locusts (*Post hoc*, Tukey test, *P* = 0.170), while there were significant differences between the isolated locusts that were injected with fluphenazine and control locusts (*Post hoc*, Tukey test, *P* = 0.010). There were also significant differences between the control gregarious locusts and the control isolated locusts (*Post hoc*, Tukey test, *P* = 0.023) (Figure 5B).

Thus the presence of the agonist caused a significant shift in the proportion of animals that exhibited a tendency to avoid the cage of locusts in the test arena, consistent with the idea that dopamine acts to drive solitarious-like behavior expressed in this paradigm. In a similar way, the injection of the dopamine antagonist fluphenazine was sufficiently efficacious to drive animals into a solitarious-like state despite being housed in a continuing crowded environment (Figure 5C). These results show that dopamine alone is sufficient to induce solitarious-like behavior in gregarious animals while the antagonist, fluphenazine, is sufficient to induce gregarious-like behavior in isolated animals. Thus, dopamine is a key neuro-active modulator of phase change during isolation.

## Discussion

Phenotypic plasticity in locusts is a complex process in which key sensory modalities are activated to drive characteristic behavior in the natural environment. Considerable insight into the biological basis of phenotypic plasticity and the formation of swarms has been achieved at the level of field investigation, however recent attention has utilized laboratory-based manipulation to mimic the environmental conditions that trigger and mediate the plasticity. These investigations have highlighted how the expression of behavioral plasticity is underpinned by physiological, neurochemical and molecular mechanisms (Matheson et al., 2004; Anstey et al., 2009; Guo et al., 2011). Much attention has focused on the changes that occur during swarm formation, but by contrast, less is known about the changes that occur in individuals during the breakdown of a swarm, or when individuals leave a group, that are as equally important in the development of new or novel control methods. As in previous studies (Roessingh and Simpson, 1994; Pener and Yerushalmi, 1998) we found that isolated locusts were less active than gregarious locusts and the transition in behavioral change was relatively short and occurred within 1 h of isolation. Interestingly in our paradigm we also observed a characteristic increased reluctance of locusts to emerge from the release chamber, consistent with a cryptic lifestyle. Roessingh and Simpson (1994) showed that this initial rapid behavioral change in response to isolation did not result in complete solitarization however, which instead was characterized by an initial rapid behavioral change, followed by a longer period that depended upon the duration of previous crowding, consistent with the changes we observed.

Physiological changes are known to accompany gregarisation in desert locusts (Matheson et al., 2004; Blackburn et al., 2010). For example, Rogers et al. (2007) showed that EPSPs evoked in FETi by a descending contralateral movement detector in gregarious locusts were half the amplitude of those in gregarious locusts, thereby reducing its excitability in gregarious animals. We found that the transition from the gregarious phase to the solitarious-like phase produced through isolation was also accompanied by significant differences in the properties of a key synapse involved in locomotory behavior, the jump. The amplitude of a CS-evoked EPSP in FETi (Burrows and Pflüger, 1988; Parker and Newland, 1995) that activates the extensor muscle during jumping, was greater and the FETi spike broader, in gregarious locusts compared to isolated animals. Physiological changes occurred at the central level (see Supplementary Figure C) and were consistent with the behavior of the two phases such that large EPSPs in gregarious locusts would make FETi more likely to spike, and therefore individuals more likely to jump (Uvarov, 1977). By contrast, a small EPSP in isolated or solitarious locusts is consistent with a reduced activity level (Roessingh et al., 1993; Simpson et al., 1999). Changes in spike duration may affect presynaptic calcium entry that regulates the amount of neurotransmitter released at a synapse (Jackson and Westlind-Danielsson, 1994; Sabatini and Regehr, 1997) whereby any increase in duration may be accompanied by an increase in excitability, as is found in gregarious behavior. Changes at the neuronal level therefore have direct impact on an animals' behavior.

### Effect of amines on synaptic transmission

While direct injection of dopamine into the thoracic haemocoel was sufficient to cause gregarious locusts to show solitarious-like behavior, it is clear from our biochemical analysis of a number of key amines in the thoracic nervous system that a suite of changes underpins phase transition. The changes we find are also consistent with previous physiological observations made against these amines that can now be discussed in the context of the biogenic amine dependence of phenotypic plasticity. Parker (1996), for example, showed that increased concentrations of octopamine, as we found in gregarious locusts, acts to mediate arousal by depolarizing flexor and extensor motor neurons controlling movements of the hind leg. Further, this was associated with a broadening of the spike in FETi, supporting the observation that gregarious locusts show greater locomotory activity. Interestingly, previous studies have shown that octopamine levels increase prior to flight (Goosey and Candy, 1980), and that it plays a key modulatory role by increasing the propensity to fly (Buhl et al., 2008). An increase in octopamine in the gregarious phase seen in this study is consistent with a role in phase change where gregarious locusts tend to fly more and are more active.

Anstey et al. (2009) showed that serotonin was both necessary and sufficient to induce gregarious behavior in desert locusts, and analysis of the levels of neurotransmitters and neuromodulators that accompany phase change showed that serotonin increases dramatically in the first 4 h of crowding of locusts (Rogers et al., 2004), supporting a central role of serotonin in mediating phase change. We also found that serotonin levels were increased on isolation of gregarious locusts. Leitch et al. (2003) showed that such an increase in serotonin levels could reduce a sensory-evoked cholinergic input onto flight motor neurons and thereby reducing their excitability, which could underpin the reduced flight activity in solitarious desert locusts.

Dopamine plays an important role in regulating and controlling many physiological and behavioral processes (Homberg, 2002). In cockroaches, it plays an important role in escape by increasing the amplitude of synaptic inputs to thoracic interneurons mediated by giant interneurons (Casagrand and Ritzmann, 1992), and is pivotal in the modulation of insect flight (Buhl et al., 2008). In *C. elegans* dopamine has been shown to control and reconfigure patterns of locomotory behavior (Chase et al., 2004; Omura et al., 2012). Other studies have shown that dopamine and serotonin can have opposite effects on behavior (Daw et al., 2002) and physiology (Claasen and Kammer, 1986; Goldstein and Camhi, 1991). This highlights the complex interaction and context-dependent role played by distinct biogenic amines raising the possibility that serotonin and dopamine might have opposing actions. Further, despite playing an essential role in initiating locust phase change it is likely that these amines alone are not the sole drivers of phase reversal given the suite of neurochemical changes found to occur, including phase related changes in a number of peptides (Claeys et al., 2006). We found that dopamine reduced the efficacy of synaptic transmission between sensory neuron and FETi, which would lead to a reduced jumping ability, and thereby mimicking the observed differences in the CS-FETi EPSP amplitude of gregarious and solitarious animals. The specificity of dopamine was confirmed by bath application of one of its antagonists, fluphenazine, that reversed the effects of dopamine at the CS-FETi synapse. Since the CS on the leg are also activated during walking, and can aid in the transition between the stance and swing phases of movement (Newland and Emptage, 1996), and also activate the SETi that receives many parallel sensory inputs to FETi (Newland and Kondoh, 1997), it likely that dopamine modulates the outputs of other components of the neural networks controlling limb movement and hence leads to the reduced locomotion found in solitarious locusts (Roessingh et al., 1993; Simpson et al., 1999). The effects of dopamine on the flight system described previously by Leitch et al. (2003) also show that this transmitter has effects consistent with it decreasing synaptic activity in specific flight motor neuron of gregarious locusts. While a functional significance for the role of dopamine on the flight system was not discussed at that time it now appears that its effects are consistent with a more general role for dopamine in orchestrating phase change and phenotypic plasticity.

### Dopamine induces solitarious behavior

Different environmental stressors such as temperature (Hirashima et al., 2000), population density (Iba et al., 1995) starvation and social interactions (Wada-Katsumata et al., 2011; Aonuma and Watanabe, 2012) all modulate the levels of biogenic amine in the central nervous system and lead insects to adapt their behavior to changing environmental conditions. Population density, in particular, leads to substantial changes in the levels of dopamine in the brains of crickets and locusts (Iba et al., 1995; Ma et al., 2011) with dopamine levels being significantly higher in solitarious migratory locusts after as little as 1 h in isolation. Rogers et al. (2004), who analyzed the entire thoracic nervous system, showed that dopamine levels increased by 3–5 fold within 1 day of isolation, but declined to low levels, typically found in gregarious locusts, following isolation for one stadium. We found that injection of dopamine into the haemocoel of gregarious desert locusts was sufficient to evoke solitarious-like behavior in gregarious animals within as little as 1 h. Unlike migratory locusts (Guo et al., 2011), however, dopamine injection into isolated desert locusts did not cause a transition to the gregarious phase, although the antagonist fluphenazine did, suggesting that endogenous dopamine may play a role in this transition. Moreover, these results clearly show that there is species-dependent modulation of phenotypic plasticity even in closely related species. These effects of dopamine parallel closely the behavioral and physiological phase changes that occur in isolated locusts and suggest that dopamine is likely to be a key modulator of locust behavior and plays a crucial role in the phenotypic plasticity underpinning this reversed phase change.

Dopamine is produced in several areas of the central nervous systems of locusts, including over 6000 neurons in the brain that are dopaminergic (Roeder, 2002). Watson (1992) found that the prothoracic ganglion had three pairs of dopaminergic somata and the mesothoracic ganglion only one pair. Watson (1992) also showed that there were dopamine immunoreactive axons in thoracic ganglia that cross into the metathoracic ganglia and also into the abdominal neuromeres, indicating a possible source of dopamine in the metathoracic ganglia. The specific role of dopamine in mediating the transition from the gregarious to the solitarious phase was confirmed *in vivo* by the injection of the dopamine antagonist, fluphenazine, which completely abolished the solitarious-like behavior of locusts isolated for 1 h. Treated locusts exhibited behavior similar to gregarious animals, implying that endogenous dopamine underpinned the change in behavior caused by isolation. It is therefore clear that in the desert locust serotonin and dopamine each play key roles in initiating different transitions of phase. It is also clear from these experiments on desert locusts that, in contrast to the migratory locust, dopamine is not the main driver of the transition from the solitary to gregarious phase suggesting that different neurochemical mechanisms are likely to underpin or differentially act upon the same transition in different species.

### Conflict of interest statement

The authors declare that the research was conducted in the absence of any commercial or financial relationships that could be construed as a potential conflict of interest.
