# Supplementary material for: Dopaminergic modulation of phase reversal in desert locusts
Source: Front Behav Neurosci. 2014 Nov 7;8:371. doi: 10.3389/fnbeh.2014.00371 (PMC4224070; doi:10.3389/fnbeh.2014.00371)
Supplement: Supplementary file 1 [file DataSheet1.PDF]

Dopaminergic Modulation of Phenotypic plasticity in Desert Locusts

Ahmad Alessi[3], Vincent O'Connor[1], Hitoshi Aonuma[2] and Philip L. Newland[1]

[1]Centre for Biological Sciences, Faculty of Natural and Environmental Sciences, University of Southampton, Highfield Campus, Southampton SO17 1BJ, United Kingdom. [2]Research Institute for Electronic Science, Hokkaido University, Sapporo 060-0812, Japan. [3]University College at Qunfudah, Umm al-Qura University, Qunfudah City, Makkah, Saudi Arabia

Supplementary Data

Reversibility of behavioral change

To analyse the reversibility of behavioral change induced by isolation, the behaviour of the same ten gregarious locusts was quantified under three conditions: long term crowded, long term crowded with 1hr of isolation, and the same locusts followed with 72 hr re-crowding. The results showed that 50% of the long-term gregarious locusts moved towards the stimulus group (n = 5) while 50% of the locusts avoided the stimulus group (n = 5)(Fig. AA).

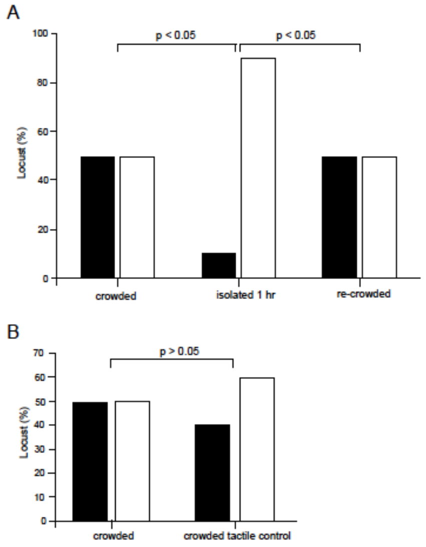

Figure A. A. Reversibility of behavioural change following 1h isolation. Behavioral analysis of 10 long-term crowded locusts that were later maintained for 1h under isolated conditions and then re-crowded for 72h showed that the change in behavior was reversible. **B.** Effect of handling on locust behavior. Statistical analysis showed that there was no significant change in behavior due to the handling process (n = 10).

All 10 locusts were then isolated for 1hr in individual cages and retested using the same assay. The behavior following 1h isolation changed compared to the control with 10% (n = 1) of the isolated locusts moving towards the stimulus group, while 90% of the locusts (n = 9) avoided the stimulus group (Fig. AA). All 10 locusts were then re-crowded for 72h and their behaviour tested again. The results showed that 50% (n = 5) of the re-crowded locusts were attracted to the stimulus group while 50% (n = 5) avoided the stimulus group. Statistical analysis showed that there were significant changes in behavior after 1hr in isolation that was reversed when locusts were re-crowded for 72hr ($\chi^2_{(1)} = 3.54$, P = 0.05, n = 10 locusts).

The effect tactile stimulation on behaviour

To control for the effects of handling and tactile stimulation impacting on behaviour a control was designed to assess their effects on behaviour. Ten locusts were taken from the gregarious colony and each locust placed individually in a cage for only 30s, after which the behavioral assay was conducted. Results showed that the handling of the locust had no effect on its behavioural choice. In the long-term gregarious group 50% of locusts were attracted to the stimulus group while 50% avoid them (n = 10). By comparison, of the handled locust controls 40% were attracted to the stimulus group while 60% avoided the stimulus group (n = 10). Statistical analysis showed there was no significant difference between two groups ($\chi^2_{(1)} = 0.180$, P = 0.67, n = 10 locusts) (Fig AB).

Biochemical analysis of four biogenic amines in the brains of individual locusts

The levels of dopamine, serotonin, octopamine and tyramine in age matched gregarious and 24hr isolated locusts were analyzed. The levels of dopamine in individual brains were significantly higher in locusts isolated for 24 hr (Mann Whitney U test, P < 0.05) (Fig. B) compared to gregarious locusts.

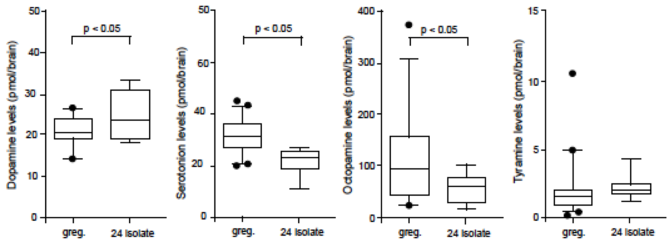

Figure B. Analysis of the levels of 4 biogenic amines in the brains of individual locusts isolated for 24 hr. Dopamine levels significantly increased in isolated locusts, whereas both serotonin and octopamine decreased (P < 0.05). The levels of tyramine in age matched gregarious and isolated locusts were not significantly different (P > 0.05).

By contrast the levels of serotonin and octopamine were significantly lower in the brains of locusts isolated for 24 hr (Mann Whitney U test, P < 0.05 for both). In common with the metathoracic ganglion tyramine showed no change in the low level of expression between long-term crowded and isolated locusts (Mann Whitney U test, P > 0.05).

Differences in CS spike activation between gregarious and solitarious locusts

The changes in synaptic strength between the CS and FETi in gregarious and solitarious locusts need not necessarily be due to central neuronal changes but could also be the result of differences in CS sensory spike activation brought about by potential changes in cuticle mechanics. Analyses were therefore made to compare CS spike activity in the two phases recorded extracellularly from N5B1. When the leg was fixed and a kick produced by ETi stimulation a burst of CS spikes was evoked with short latency following stimulation of ETi (Fig. CA). A similar amplitude of spikes was evoked during manual deflection

of the tibia to the spikes evoked during a kick indicating that the spikes evoked during a kick were from the anterior CS.

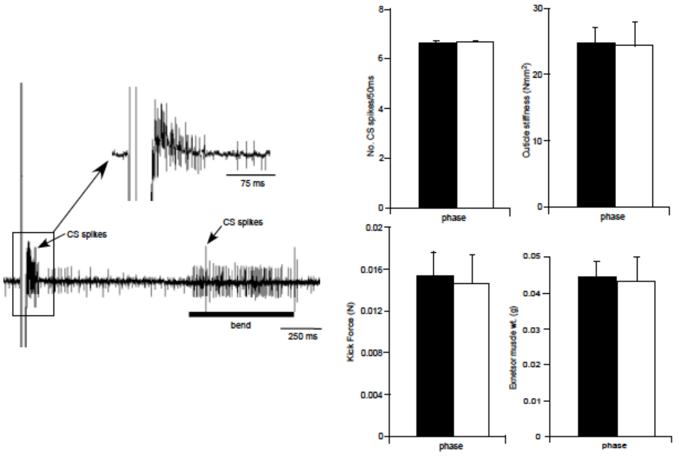

Figure C. Control experiments. **A.** Spikes of CS recorded from N5B1 were evoked during a kick and during deflection of the cuticle of the proximal tibia. **B.** The number of spikes evoked in the CS was not significantly different between phases. **C.** There was no significant difference in the bending stiffness of the cuticle of solitary and gregarious locusts the two phases (Student's T-test, P = 0.941). **D.** There was no significant difference in kick force between the two phases (Student's T-test, P = 0.66) evoked by stimulating ETi. **E.** Comparison of the weight of the extensor muscles in gregarious and solitarious locusts. There were no significant differences between the two phases (Student's T-test, P = 0.895).

To compare CS spike activity in the two phases the number of CS spikes evoked was counted in a 50 ms window following the artifact caused by the stimulus to produce a kick. The number of CS spikes evoked in N5B1 was 6.70 ± 0.14 in gregarious locusts and 6.70 ± 0.26 in solitarious locusts (Fig. CB). A Student's unpaired T-test showed that there was no statistical difference between the numbers of spikes evoked in the two phases (n = 6, P = 1.000).

Comparison of cuticle stiffness, kick force, extensor muscle weight between phases

Cuticle Stiffness. To control for cuticle stiffness causing a difference in CS-FETi EPSP amplitude between phases a comparison of the cuticle stiffness in solitary and gregarious locusts was carried out. An approach was developed to quantify the stiffness of the cuticle. Ten 3d age-matched gregarious and ten solitarious locusts were used to compare cuticle stiffness between phases. The stiffness of the cuticle was 24.33 ± 3.34 Nmm^2 (n = 10) in solitarious locusts, and 24.69 ± 2.29 Nmm^2 in gregarious locusts (n = 10), and statistical analysis revealed that, there was no significant difference in cuticle stiffness between the two phases (Students T-test, P = 0.941) (Fig. CC).

Kick force. Since the degree of bending of the tibia is dependent on the cuticle stiffness, the force generated by the kick could vary, and in turn cause differential activation of the CS leading to differences in the FETi EPSP amplitude between the phases. The force of the kick was, therefore, measured and compared between gregarious and solitarious locusts. Adult locusts were fixed ventral-side-up on a clay platform using modeling clay. The left hind leg was fixed with the tibia and tarsi of the leg free to move within their full range. The force of the kick was recorded five times for each locust and at the end of recording a calibration was carried out by suspending a 500 mg weight from the load cell. The force generated during a kick was calibrated using the equation $F = M \times A$, where F was the resulting force of a kick, M was the mass of the calibration weight, and A was its acceleration.

The extensor muscle of a hind leg was stimulated to evoke a kick and the force of the kick measured using the load cell. The mean force of the kick was 0.0146 ± 0.0028 N (mean ± SEM) in solitarious locusts (n = 18), and 0.0154 ± 0.0022 N (mean ± SEM) in gregarious locusts (n = 18), however, the difference in the means of the two phases was not significantly different (Student's T-test, P = 0.669) (Fig. CD).

Extensor Muscle mass. The mass of the extensor tibia muscle could also have an effect on the force of kick that may in turn lead to differences in the EPSP amplitude between the solitary and gregarious phases. The mass of the extensor muscle of solitarious and gregarious locusts was therefore compared. Extensor muscles were removed by dissection and results showed that the mean wet weight of the muscle was 0.039 ± 0.006 g in solitarious locusts (n = 17) and 0.040 ± 0.004 g in gregarious locust (n = 17) (Fig. CE). There was no statistically significant difference between the two phases (Student's T test, P = 0.895).

Effect of dopamine on FETi EPSP amplitude in solitarious locusts

To determine whether dopamine also had an effect on the modulation of synaptic transmission between CS and FETi in solitarious locusts, gregarious locusts were isolated for 1hr under standard isolated conditions and FETi recorded and ETi stimulated to evoke a CS-evoked compound synaptic potential in FETi. The protocol for the experiment was similar to that used for gregarious locusts. The results showed that the EPSP amplitude in solitarious locusts was also reduced considerably after 5 min of 10mM dopamine perfusion (Fig. DA).

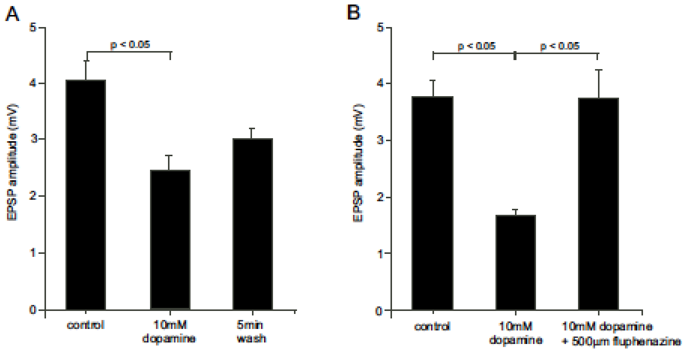

Figure D. The effect of dopamine on a CS-evoked EPSP in solitarious locusts. **A.** Dopamine caused a decline in the EPSP amplitude after 5 min of application and the effect was reversed after a 5 min wash in normal saline. There was a statistically significant decrease in EPSP amplitude under dopamine (10 mM) conditions compared to control (One way ANOVA, $F_{2,16} = 5.07$, $p < 0.05$). n = numbers of animals tested. **B.** The effect of co-application of dopamine and fluphenazine on EPSP amplitude in gregarious locusts. Bath application of dopamine (10 mM) led to a reduction in EPSP amplitude within 5 mins. The simultaneous co-application of dopamine and fluphenazine (500 µM) led to a recovery of EPSP amplitude to control levels.

The EPSP amplitude under control conditions was 4.05 ± 0.37 mV and decreased significantly to 2.47 ± 0.27 mV after 10 mM dopamine perfusion for 5 min (One way ANOVA, $F_{2,16} = 5.07$, $p < 0.05$). The amplitude recovered to 3.01 ± 0.22 mV, after washing with standard locust saline for 5 min and was not significantly different from control amplitude.

Fluphenazine antagonizes the effects of dopamine

Further analysis to test whether fluphenazine antagonizes the decrease in EPSP amplitude in FETi caused by dopamine was

carried out by applying dopamine and fluphenazine in the same experiment to gregarious locusts. Standard saline was initially perfused continuously into the locust thorax and CS-evoked EPSPs in FETi recorded intracellularly. 10 mM dopamine was then perfused into the locust thorax for 5 min which caused a decrease in the CS-evoked EPSP amplitude after 5 min of application, as was also shown earlier. Fluphenazine (500 μM) was then perfused, simultaneously with 10mM dopamine into the thorax and was found to cause an increase in EPSP amplitude, returning it to control levels (Fig. DB).

Effect of chlorpromazine (CPZ) on EPSP amplitude

Chlorpromazine (CPZ) is an effective antagonist of dopamine, which is selective for all dopamine receptors (Degen et al., 2000a). Chlorpromazine was therefore perfused into the locust thorax continuously to block the action of dopamine that caused a decrease in the amplitude of the CS-evoked EPSP in FETi. 1mM chlorpromazine caused a steady, non-reversible, reduction in EPSP amplitude over 5 mins (Fig EC). The reduction in EPSP amplitude began immediately after application of the drug and completely abolished the EPSP within 5 min (Fig EA). There was a statistically significant, non-reversible, effect of concentration on EPSP amplitude (One way ANOVA, $F_{30, 270} = 22.5$, $p < 0.05$).

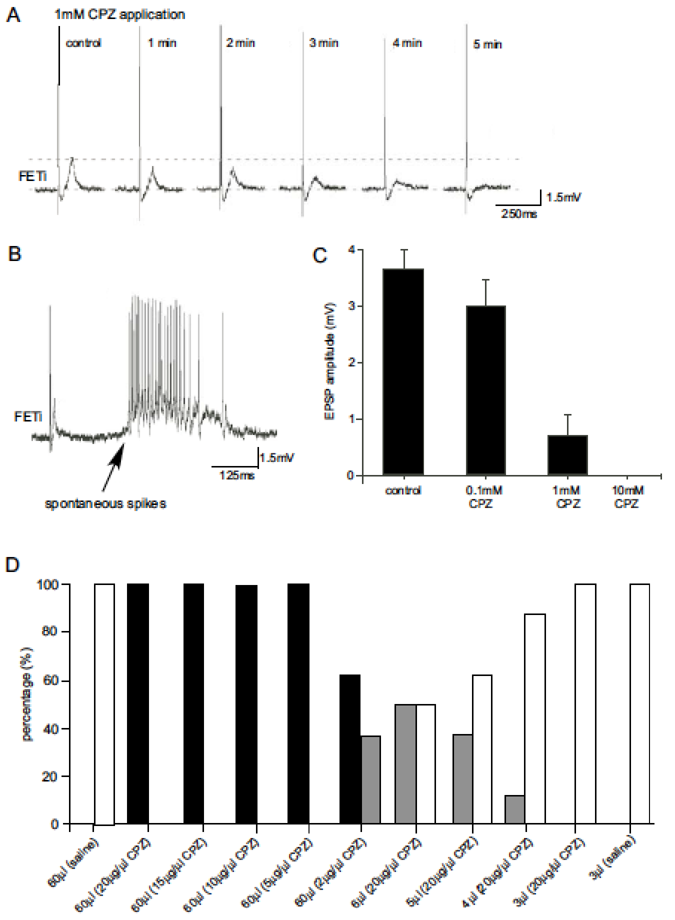

Figure E. The effect of CPZ on synaptic transmission between CS and FETi and locust viability. **A.** Recording of FETi showing the effect of CPZ (1 mM) on EPSP amplitude over a 5 min recording period. 1 mM CPZ caused a gradual decrease in EPSP amplitude until abolishing it after 5 min. **B.** The application of 10 mM CPZ caused a rapid burst of spikes in FETi followed by a complete lack of spike or synaptic activity. **C.** Graph showing the effects of concentration (0.1 mM, 1 mM and 10 mM) on synaptic transmission. The EPSP amplitude decreased dramatically after the application of CPZ (0.1 mM, 1mM and 10 mM). A high concentration of CPZ (10 mM) abolished the EPSP (n = 13, 5, 5 and 5locusts respectively). **D.** The effect of different concentrations of CPZ (20, 15, 10, 5 and 2 μg/μl), and different injected volumes, on locust viability. 3μl (20 μg/μl) was the highest amount of CPZ that had no overt effect on the locusts tested and was used for subsequent behavioural analysis.

Results showed that relatively high concentrations of CPZ (10 mM) caused rapid bursts of spikes in FETi followed by a complete abolishment of spike and synaptic activity (Fig EB). A concentration series of CPZ (0.1 mM, 1 mM and 10 mM) was therefore used to determine if lower concentrations had any effect on the EPSP amplitude (Fig EC).

The effect of chlorpromazine on behavior

Dopamine had the effect of changing the choice behavior of locusts. To test the specificity of dopamine the effect of the dopamine antagonist, chlorpromazine(CPZ), on choice behavior was examined. Gregarious and solitarious locusts were injected with a solution of CPZ (3 μl, 56 mM, Ma et al., 2011) and their behaviour analyzed after 1hr. Preliminary observations, however, showed that there were deleterious effects of the injection of CPZ on the locusts, with some animals dying and others becoming quiescent after the treatment (Fig. EE). A concentration series of CPZ (20, 15, 10, 5 and 2 μg/μl) and different volumes (60, 6, 5, 4 and 3 μl) was therefore used to test its effect on locust activity. 8 locusts were injected with CPZ at each concentration and 8 locusts were injected with saline. The choice behaviour of locusts remaining 1 hr after CPZ treatment was then assayed (see below). Injection of 60 μl (20, 15, 10 and 5 μg/μl) CPZ caused death of all locusts after 1 hr. By contrast, the injection of 60 μl normal saline caused neither death nor resulted in inactivity of any animal. 60 μl (2 μg/μl) CPZ caused death in 63% of locusts while the remaining 37% remained alive but inactive. The injection of 6, 5 and 4 μl (20 μg/μl) CPZ did not lead to death of any locusts but caused inactivity in many, with the amount of inactivity decreasing with lower concentrations of CPZ. However, the injection of 3μl (20 μg/μl) CPZ caused neither death nor inactivity in any animal, similar to the injection of 3μl saline. 3μl (20 μg/μl) CPZ was therefore found to be the highest concentration of CPZ that had no apparent detrimental effect on the locusts. Gregarious and solitarious groups were injected with 3μl (20 μg/μl) CPZ, and control groups injected with the same volume of saline (3μl), and both groups rehoused within their respective colonies for 1 hr. Individual locusts were then released into the test arena for analysis of their choice behavior within a 10 min period. Results showed that CPZ had no effect on the behaviour of either gregarious or solitarious locusts. Behavioural analysis showed that 65% (n = 32) of the control gregarious locust were attracted to the stimulus group and 35% (n = 17) avoided them, while gregarious locusts treated with CPZ showed similar behavior, with 67% (n = 16) attracted to the stimulus group and 33% (n = 8) avoiding them, indicating that the concentration that has no impact on the animal health had no effect on behavioral choice.
